# History of Arbovirus Research in the Czech Republic

**DOI:** 10.3390/v13112334

**Published:** 2021-11-22

**Authors:** Zdenek Hubálek

**Affiliations:** Institute of Vertebrate Biology, Czech Academy of Sciences, Květná 8, 60365 Brno, Czech Republic; zdehubalek@seznam.cz

**Keywords:** arthropods, ticks, mosquitoes, mammals, birds

## Abstract

The aim of this review is to follow the history of studies on endemiv arboviruses and the diseases they cause which were detected in the Czech lands (Bohemia, Moravia and Silesia (i.e., the Czech Republic)). The viruses involve tick-borne encephalitis, West Nile and Usutu flaviviruses; the Sindbis alphavirus; Ťahyňa, Batai, Lednice and Sedlec bunyaviruses; the Uukuniemi phlebovirus; and the Tribeč orbivirus. Arboviruses temporarily imported from abroad to the Czech Republic have been omitted. This brief historical review includes a bibliography of all relevant papers.

## 1. Introduction

The investigation of arboviruses and arboviral diseases has a long history in the Czech lands. It started in 1948, and the first papers were published in 1949 [1,2,3,4,5,6]. Basic research of arthropod-borne viruses (with the acronym “arboviruses”) has been mainly organized at three institutes in the Czech lands: the Institute of Parasitology (Czech Academy of Sciences, Prague and České Budějovice), the National Institute of Public Health (Prague) and the Institute of Vertebrate Biology (Czech Academy of Sciences, Brno). However, a number of other laboratories (e.g., the Regional Hygiene Station in Ostrava and some other district public health stations) and researchers also contributed significantly to the study of arboviruses in the Czech Republic. The following description of arbovirus studies covers the papers published between 1948 and 2021. The papers have been included in this review if they investigated arboviruses occurring in the Czech lands or they described laboratory studies carried out by Czech virologists focusing on such arboviruses. Arbovirus infections temporarily imported from abroad have not been included, nor have the field studies of Czech arbovirologists abroad.

Arboviruses belong to an ecological group of viruses characterized by their specific biological transmission via competent hematophagous arthropods (e.g., ticks, mosquitoes and others) to homeotherm (warm-blooded) vertebrates. Competent vectors are those arthropods that are able to imbibe the virus in the course of blood-feeding on an infected donor vertebrate host to support the multiplication of the virus in their organism and to deliver a sufficiently large inoculum to the recipient (i.e., the uninfected vertebrate host). Usually, a certain minimum level of viremia (i.e., the “infection threshold”) in a donor vertebrate host is necessary for efficient infection of particular arthropod vectors. Therefore, only those vertebrate species that produce at least moderate viremia have been regarded as competent, “true” or “amplifying” hosts of particular arboviruses [7]. However, co-feeding of the ixodid ticks on a viremia-free host can sometimes also contribute to the infection of noninfected ticks. Some arboviruses are transmitted from larvae to the nymphs and imagoes of arthropods during metamorphosis (i.e., transstadial transmission (TST)), from infected females to the offspring (i.e., transovarial transmission (TOT)) and from males to females during copulation (i.e., venereal or horizontal transmission). These transmission modes are important ecologically. For example, under the conditions of TOT, the vector also plays the role of a long-term reservoir of the virus in nature.

In addition to a few severe, occasionally re-emerging viral diseases transmitted by ixodid ticks or mosquitoes in Czech lands (tick-borne encephalitis and West Nile fever), there are a number of other usually neglected arbovirus infections of vertebrates which are infrequent, likely underdiagnosed and non-pathogenic for humans.

Several monographs about multiple arboviruses in Czechoslovakia were published [8,9,10,11,12,13,14]. Particular arboviruses are treated separately in the following text.

## 2. Family: *FLAVIVIRIDAE*

### 2.1. Tick-Borne Encephalitis Virus (TBEV; Genus: Flavivirus)

The history of TBE in Czech lands and research on TBE is a long-lasting story that is very rich and dramatic. The first cases of TBE were diagnosed in several districts (Beroun, Strakonice, Nový Bydžov and Vyškov) in the summer of 1948 [1,2,4]. For instance, 56 TBE patients were found in the Vyškov district in 1948 and 22 patients in 1949 [15]. TBEV was isolated from the blood and CSF of several patients from the districts of Beroun [1], Strakonice [2,3], and Vyškov in the summer of 1948 [4,5]. The original virus isolate (strain “Stillerová”) was found [16] to be closely related to the louping ill virus (LIV) and Russian spring-summer encephalitis virus (RSSEV) by cross-VNT, cross-CFT and cross-protection, although it is more pathogenic for laboratory mice than LIV.

The characteristics (e.g., passage history, disease in mice and guinea pigs, neutralization assays and ultrafiltration) of the isolated TBEV strains were described in detail [1,17]. Krejčí [4] recorded that 74% of his patients in the Vyškov area were attacked by *Ixodes ricinus* ticks (e.g., during mushroom collections) in local forests. Inspired by this notice, the successful isolation of TBEV from ixodid ticks was then reported in the Beroun [6] and Vyškov areas in 1949 [18]. TBE cases also occurred in the Vyškov and Strakonice areas in the following years [15,19,20,21,22,23,24,25,26], as well as in the lower Sázava river valley [27] and Brno region [28,29]. Additional TBEV strains were isolated from patients and ixodid ticks in Bohemia [30,31] and in the Brno region, including the prototype TBE strain “Hypr” [31,32].

Some physicians [33,34] drew attention to certain non-diagnosed cases of aseptic meningoencephalitis in the Czech Republic before the Second World War, being convinced that some of them were, in fact (according to the described clinical symptoms), most probably TBE, such as those reported in 1924–1932. Retrospectively, TBE has been detected by serology in forest workers in the Brno region since 1939 [35].

The Czech TBEV strains have thus been regarded as closely related to LIV and RSSEV [16]. The close relationships of the Czech TBEV, Russian TBEV (RSSEV) and LIV were confirmed [36,37], and the first electron microscopy pictures of the Czech TBE were published [38]. Much later, it was reported [39] that Central European TBEV strains are antigenically very homogeneous and closer to LIV than to RSSEV. Because the Russian strains are now regarded as a variant of the TBEV species, LIV should be considered as a variant within the same TBEV species. Subtyping of the TBEV isolates using multiplex RT-PCR was suggested [40].

The principal arthropod vectors are ticks of the genus *Ixodes:* for TBEV *I. ricinus* in central Europe (TST and TOT confirmed [41,42]), and the prevalence rate of TBEV in *I. ricinus* populations may reach 0.5–3% in the valent natural foci [43,44]. Occasional vectors are other tick species such as *I. hexagonus* [45] and possibly *I*. *arboricola*. The TOT of TBEV in ticks [46,47] is epidemiologically very important in that it makes possible the long-term persistence of the virus in a natural focus. Mosquitoes and fleas are not vectors of TBEV, as was proven experimentally [48,49,50].

In experiments, the relative humidity affected the infection rate of TBEV in *I. ricnus* ticks, but the temperature did not [51,52]. The interaction of a temperature-sensitive mutant with a virulent TBEV strain was observed in the experimental dual infection of *I. ricinus* ticks [53], where the mutant partially inhibited the replication of the virulent strain.

TBEV multiplied in all the tested cell lines derived from diverse ixodid ticks, but CPE was not observed [54]. However, in cell lines derived from the vector tick species (*I. ricinus*), there was a 100–1000-fold higher virus yield than in the cell lines derived from non-vector ixodid ticks (*I. scapularis*, *Boophilus mocrolus*, *Hyalomma anatolicum*, etc.). The differences between the TBEV grown in mammalian and tick cell lines were studied [55,56]. Mapping of the envelope protein of TBEV grown in parallel in human and tick cells also revealed some differences between the two lines [57]. The question of whether two different cell lines (from *I. scapularis* and *I. ricinus* ticks) responded to infection with TBEV in the same way was investigated by transcriptomic and proteomic analysis [58]. Several molecules were identified that may be involved in the tick cells’ innate immune response against flaviviruses.

The competent vertebrate hosts of TBEV are certain mammals, especially rodents and insectivores such as *Apodemus flavicollis*, *A. sylvaticus*, *Myodes glareolus*, *Microtus agrestis*, *Sciurus vulgaris* [59], *Talpa europaea* [60,61], *Sorex araneus* and *Erinaceus concolor*, as well as goats [62], sheep and rarely cattle. However, the so-called non-viremic transmission of TBEV by co-feeding of non-infected and infected ticks on incompetent vertebrate hosts could also be important in natural foci [63,64,65]. TBEV was isolated from the brain of *A. sylvaticus* and *M. glareolus* in the North Moravian region in 1956–1959 but not from rodents of the other seven rodent spp. and *S. araneus* [66]. However, Kožuch et al. [67] isolated TBEV from *S. araneus* in the same region. The TBEV seropositivity in four spp. of bats in Czech lands was reported [68,69], though the ecological interpretation of this finding is difficult. Experimental infection of several *Myotis myotis* bats with TBEV [70] resulted in viremia (up to 12 DPI), and the virus was isolated from the visceral organs and brain at up to 12 DPI. Some bats showed paresis or even quadriplegia. High titers of antibodies to TBEV were detected by CFT in forest rodents, deer (*Cervus elaphus* and *Capreolus capreolus*), hares and wild rabbits, *Sciurus vulgaris* and *Meles meles* in the natural focus near Beroun [31,59]. Laboratory mice and rats, including adult ones, are very susceptible to TBEV and die within few days after i.c., s.c., i.p. or i.n. inoculation with the virus [1,4,5,17,18,42,71,72,73,74]. The interaction of TBEV with mouse peritoneal macrophages was described and modified by antibodies and lectin [75]. Experimental s.c. infection with TBEV in wild bank voles (*Myodes glareolus*) and laboratory mice [76] resulted in survival of the voles (contrary to white mice), and they had higher viral titers in the regional lymph nodes but much lower viral load in the brain. In addition, the TBEV infectivity for *M. glareolus* and *A. flavicollis* from two habitats was tested [77]. TBEV infection was compared in laboratory mice and wild *Apodemus* field mice, in which the latter survived and had no virus present in the brain, higher NK cell activity, no multiplication of TBEV in the peritoneal macrophages and higher titers of VN antibodies. Both species revealed viremia and viral presence in the spleen [78]. In one *M. glareolus* examined from a military area in central Bohemia, TBEV RNA was detected in the visceral organs [79]. Experimental TBEV viremia was demonstrated in many mammalian, avian, amphibian and reptilian species, such as in bats [70], *Talpa europaea* [60,61,80], *Microtus subterraneus* [81], *Micromys minutus* [82,83] and some other vertebrate species. The monkeys *Macaca mulatta* and *M. fascicularis* (=*M. cynomolgus*) were killed by TBEV [84,85], except when inoculated by the respiratory route [86]. However, no clinical signs in rhesus monkeys were observed when inoculated s.c. with high doses of TBEV, but viremia (3–11 DPI) and antibodies (VN and CF after 2–3 weeks) were present [86,87].

Kolman and Husová [88] examined small mammals (136 *Apodemus flavicollis*, 111 *Myodes glareolus* and 127 *Microtus arvalis*) in a natural focus at Unhošť in central Bohemia via the HIT. Antibodies to TBEV were present in the three species at rates of 0.7%, 1.8% and 4.5%, respectively. The seropositivity rate (HIT) in 84 examined forest rodents (*Myodes glareolus* and *Apodemus* spp.) in the Bohemian Krušné hory mountains was 9.5% [89], and seropositivity was found in the wildlife and small wild mammals in south Moravia via the HIT [90].

The role of birds as hosts of TBEV has not yet been fully elucidated, as the virus has been occasionally isolated from some passeriform and other birds. For instance, 809 wild birds were shot in southern Bohemia and examined. One strain of TBEV was isolated from *Fulica atra* coot, and VN antibodies to TBEV were detected in one each of the stork *Ciconia ciconia* and pheasant *Phasianus colchicus*. Only 1.7% of the birds were parasitized by ixodid ticks [91,92]. In 13% of 338 wild birds (mostly passerines) shot in central and eastern Bohemia, antibodies to TBEV were detected [93]. However, heart extracts collected from the shot birds could contain non-specific virus-inhibiting compounds (e.g., traces of bile). One teal (*Anas crecca*) of the 84 examined wild ducks and geese (6 spp.) in the Czech lands was seropositive for TBEV by VNT but at a very low titer of 1:8 [94]. Juřicová et al. [95] tested via the HIT 295 passeriform birds caught in south Moravia, and TBEV antibodies were present in 7.1% of them. Four of 82 passerine birds (14 spp.) in the Krkonoše mountains netted during the autumn migration contained HI antibodies to TBEV [96]. In 273 house sparrows (*Passer domesticus*) caught in northern Moravia and tested for their HI TBEV antibodies were found in 1.8% of them [97]. The chick embryos were killed by inoculation of TBEV [98,99,100,101,102,103,104].

TBE in domestic animals has not been fully investigated [105]. TBEV infection is usually subclinical in adult ruminants, and pigs, goats, sheep and cows excrete the virus in their milk. In dogs, several cases of TBE were described, including three dogs which had meningoencephalitis [106].

Humans acquire TBEV infection through the bite of an infectious tick or by consumption of infected raw (unpasteurized) goat or sheep (less often cow) milk or dairy products [107,108,109,110,111,112,113,114]. Tick-transmitted cases tend to be sporadic, while milk-borne infections usually affect whole families or population groups in outbreaks. For instance, one very large milk-borne TBE epidemic occurred in Rožňava, East Slovakia in 1951, when 660 persons were infected and 274 of them were hospitalized. In addition, many laboratory infections (usually occurring due to infectious aerosols) have been reported in unvaccinated personnel [1]. TBE could present an occupational risk in foresters or farmers, since these persons have a greater risk of exposure to ticks [115,116].

Hanzal and Henner [117,118] differentiated four clinical forms of TBE in humans: abortive, meningeal, encephalitis and encephalomyelitis. The clinical forms of TBE were described by many other authors in the Czech lands, as well as the neurologic sequelae [4,15,21,119,120,121,122,123,124,125,126,127,128,129,130,131,132,133,134,135,136,137,138,139,140,141,142,143,144,145,146,147,148,149,150,151,152,153,154]. The pathogenesis of TBE was studied by a number of Czech authors [155], and the histopathological changes in the mouse brain, typical for encephalitis, were reported [156,157,158]. Jandásek [159,160] observed the blood–brain barrier crossing of TBEV in a mouse model. Kašová-Odrážková [161] detected antibodies in the CSF of TBE human patients. Málková et al. [162,163,164,165,166,167,168,169,170,171,172,173,174] and Málková and Filip [175] confirmed that the lymphatic system is an important route for TBEV dissemination in the bodies of experimental animals. The histopathological changes in monkeys infected by TBEV were studied, and the changes were similar to those in humans [176]. Several authors [177,178] observed modulation of the changes of infected dendritic cells caused by tick saliva. Cultured mouse dendritic cells were infected with two TBEV virus strains of different virulences in the presence of *I. ricinus* tick saliva. Tick saliva treatment increased the proportion of virus-infected cells, leading to a decrease in virus-induced TNF-alpha and IL-6 production and to reduced virus-induced apoptosis, which may have positive consequences for TBEV replication and transmission. The effect of TBEV on the morphology human neural cells was also studied in detail. Three human neural cell lines (neuroblastoma, medulloblastoma and glioblastoma) were infected with TBEV, and the neural cells produced roughly from 100- to 10,000-fold more virus titers than the conventional cell lines of extraneural origin, indicating the highly susceptible nature of neural cells to TBEV infection. The infection of medulloblastoma and glioblastoma cells was associated with a number of major morphological changes, including proliferation of the membranes of the rough endoplasmic reticulum and extensive rearrangement of cytoskeletal structures. Cells were dying preferentially by necrotic mechanisms rather than apoptosis [179,180,181,182]. In another study, the host cell receptor for TBEV was identified by a complex procedure. Anti-idiotypic monoclonal antibodies (anti-ID MAbs) were formed against two mouse MAbs that neutralized the infectivity of TBEV. Three of the anti-ID MAbs inhibited the binding of the respective idiotypic MAb to the TBEV antigen and bound to the surfaces of virus-susceptible (but not non-susceptible) cells. They recognized a 35-kD protein in the immunoblotting analysis [183]. The growth of TBEV in cultured mouse macrophages was tested, and the virus replication process differed from that in other mammalian cell lines, since the smooth membrane structures, which are thought to be the sites for flavivirus replication, were not observed. Moreover, different TBEV strains exhibited different interactions with the host macrophages [184]. Palus et al. [185,186] demonstrated injury to human astrocytes by TBEV and novel inflammatory markers in TBE pathogenesis. CD8+ T-cells have been shown to mediate immunopathology in TBE, as demonstrated by the prolonged survival of SCID or CD8(-/-) mice following infection when compared with immunocompetent mice or mice with adoptively transferred CD8(+) T-cells. The results imply that the inflammatory reaction significantly contributes to the fatal outcome of TBE [187]. The blood–brain barrier (BBB) integrity and inflammation in the CNS of TBEV-infected animals were studied by several authors [188,189,190]. For instance, primary human microvascular endothelial cells (HBMECs) were infected with TBEV to study their interactions with the BBB. The infection was persistent, with high TBEV yields. Infection did not induce any significant changes in the expression of key tight junction proteins and did not alter the highly organized intercellular junctions between HBMECs. In this in vitro model, the virus crossed the BBB via a transcellular pathway without compromising the integrity of the cell’s monolayer. The results indicate that HBMECs may support TBEV entry into the brain without altering the BBB integrity. The authors also followed serum metalloproteinases in 147 TBE patients. Matrix metalloproteinase-9 (MMP-9) and tissue inhibitor of metalloproteinase-1 (TIMP-1) play important roles in the function of the blood–brain barrier (BBB), and they were measured by ELISA in serum from patients with an acute phase of TBE. The MMP-9 levels and MMP-9/TIMP-1 ratios of the TBE patients were significantly higher than those of the controls. The results suggest that the increased serum level of MMP-9 and the MMP-9/TIMP-1 ratio is associated with the pathogenesis of TBE. Serum MMP-9 can serve as an indicator of breakdown of the BBB and inflammatory brain damage during TBE [191]. A novel locus on mouse chromosome 7 affects the survival of the host infected with TBEV [192]. Changes in the cytokine and chemokine profiles in the mouse serum and brains of infected mice were investigated [193]. TBEV-infected mice exhibited increases in serum and brain tissue concentrations of multiple cytokines and chemokines (mainly CXCL10/IP-10, as well as CXCL1, G-CSF, IL-6 and others). TBEV-infected SK-N-SH cells exhibited increased production of IL-8 and downregulated MCP-1 and HGF. TBEV infection of HBCA cells activated the production of a broad spectrum of pro-inflammatory cytokines, chemokines and growth factors (mainly IL-6, IL-8, CXCL10 and G-CSF) and downregulated the expression of VEGF. Treatment of SK-N-SH cells with supernatants from infected HBCA-induced expression of a variety of chemokines and pro-inflammatory cytokines reduced the SK-N-SH mortality after TBEV infection and decreased virus growth in these cells. Treatment of HBCA with supernatants from infected SK-N-SH cells had little effect on cytokine, chemokine or growth factor expression but reduced TBEV growth in these cells after infection. The results indicated that both neurons and astrocytes are potential sources of pro-inflammatory cytokines in TBEV-infected brain tissue. Infected or activated astrocytes produce cytokines or chemokines that stimulate the innate neuronal immune response, limiting virus replication and increasing the survival of infected neurons.

When TBEV was passaged serially in either mice or PS (pig kidney) cells at 40 °C, the authors obtained a new variant strain with increased plaque size, invasiveness and temperature resistance and with two unique amino acid substitutions. They explained this as a process of selection of pre-existing virulent variants in the TBEV gene pool and not as the appearance of a new mutant [194]. The effect of passaging TBEV in different host cell systems was also observed by other investigators [195]. A highly virulent strain (Hypr) of TBEV was serially subcultured in the mammalian porcine kidney stable (PS) and *I. ricinus* tick (IRE/CTVM19) cell lines, producing three viral variants. These variants exhibited distinct plaque sizes and virulence in a mouse model. When comparing the full-genome sequences of all variants, several nucleotide changes were identified in different genomic regions. Furthermore, different sequential variants were revealed to coexist within one sample as quasispecies. These observations further imply that TBEV exists as a heterogeneous population that contains virus variants pre-adapted to reproduction in different environments (ticks and mammals).

Human incidence of TBE in particular areas of the Czech lands was reported in a number of papers [196,197,198,199,200,201,202,203,204,205,206,207,208,209,210,211,212,213]. The incidence rate is relatively high in the Czech lands compared with other European countries. On average, 368 cases (140–744 in individual years) per year were reported in the Czech Republic between 1970 and 1999 with a morbidity rate of 4.2 (1.4–7.4) per 100,000 inhabitants, and it peaked at 1029 patients with a morbidity rate of 10.0 per 100,000 inhabitants in the year 2006 [214,215]. In the years 2004–2007, only Slovenia and the Baltic republics (Estonia, Lithuania and Latvia) had higher morbidity rates of TBE than the Czech Republic (5–10), while the TBE incidence was as low as 0.6–1.2 in neighboring Austria due to a much higher vaccination frequency in that country. Vaccination rates against TBE in the human population in the Czech lands are roughly 15% [205].

The natural foci of TBE are classified according to the hosts of the principal vector as “theriodic” (i.e., situated in deciduous and mixed forest ecosystems with wildlife, often in game preserves), “boskematic” (i.e., pastoral, with pastured domestic mammals), mixed “theriodic-boskematic” or “mountain” [216]. The periurban foci of TBE have also been described [217,218,219]. The foci were analyzed for their original plant (forest) communities in different areas of the Czech lands [220,221,222] and in the vertebrate fauna [223,224]. The use of satellite data (remote sensing) may be helpful for the risk assessment of areas with high populations of ixodid ticks, which are vectors of TBEV [225,226,227]. The natural foci of TBE have been studied in different parts of Bohemia [31,88,228,229,230,231,232,233,234,235,236,237,238,239] and Moravia [67,235,240,241,242,243,244,245,246,247,248,249,250,251,252].

During the last three decades, a significant increase in the incidence of human TBE has followed a shift of *I. ricinus* ticks to higher altitudes (above 700 m a.s.l.) in the Bohemian and Moravian mountains (Šumava, Krkonoše, Jeseníky and Czech-Moravian Highland), likely in response to climate warming [253,254,255,256,257,258,259,260,261,262,263,264]. The effect of the weather and climate on the TBE incidence has been demonstrated [211,214,215,257,259,263,264,265,266,267,268,269,270,271,272,273]. Positive correlation between the daily mean air temperature and lagged (by 3–11 days) human TBE incidence (a total of 4613 cases recorded) in the Czech Republic was found for the years 1994–2001 [264,267]. The authors explained this phenomenon with the increased host-seeking activity of *I. ricinus* tick vectors and human behavior, in that TBE in the Czech Republic is largely a recreation-based disease connected with human outdoor activities [274]. No correlation was found for precipitation. Significant correlation between the enhanced rodent population size and increased human TBE incidence in a following (lagged) calendar year was found by several authors [275,276,277,278,279].

TBEV strains in central Europe are regarded as antigenically stable and genetically homogeneous [39]. The isolation of four temperature-sensitive (t_s_) small plaque mutant TBEV strains from *I. ricinus* collected in the natural foci of south Bohemia is of special interest, because these mutants are markedly less pathogenic for s.c. inoculation into adult mice than typical TBEV strains. These mutants caused complete protection against challenges with “normal” TBEV strains such as Hypr. Moreover, the t_s_ mutant also interfered with the Hypr strain and blocked its replication in female *I. ricinus* ticks in experiments [280].

TBEV has been studied in cell cultures in vitro [281]. CPE and distinct plaques of TBEV are produced in porcine embryonal kidney cell lines PK, PS and SPEV [282,283,284] and in CV-1 monkey cells [285,286,287], which are highly applicable for VNT and PRNT.

Many Czech virologists contributed to the serological diagnosis of TBE [288,289,290,291,292,293,294,295]. Acute TBE can be diagnosed through the detection of IgM antibodies using indirect IFA [296,297,298]. The preparation of hyperimmune mouse ascitic fluids and IFA were described as a useful tool for the identification and serodiagnostics of TBEV [299,300]. For rapid serodiagnosis, ELISA with purified TBEV antigen from infected mouse brains can be used [301,302]. Monoclonal antibodies to TBEV were prepared for the identification and antigenic differentiation of TBE complex viruses [303,304,305,306]. The monoclonals were also used for the analysis and localization of TBEV antigens, especially glycoprotein E and non-structural protein NS3 [305]. The cryo-EM structures of native TBE virions and their neutralization by monoclonal antibodies (its complex with Fab fragments of the antibody) was visualized [306].

Heinz et al. [307] compared the sensitivity of chick embryo cells and young mice for the isolation of TBEV, and the mice were more susceptible. Different methods for the detection of TBEV or its RNA in various specimens were reviewed [308,309]. Genome sequencing of TBEV isolates from patients was proposed as epidemiologically helpful [310].

The prevention and control of TBE include mapping and surveillance of their natural foci [221,222,225,226,265,269,311] and immunization of humans. Vaccines against TBEV consist of a purified, inactivated virus grown in chicken embryo cells produced by methods largely based on the original studies of several Czech virologists [312,313,314,315,316,317,318,319,320,321,322,323]. In the Czech Republic, two vaccines against TBE are presently registered: FSME IMMUN (Baxter) and ENCEPUR (Behringwerke). These vaccines were tested for immunogenicity, tolerance and antibody persistence in this country [324,325,326,327,328,329]. A TBE vaccine has also been developed for domestic animals [330,331].

For therapy of TBE, cortisone and corticoids could be helpful [150,332,333]. Specific immunglobulin or human antiserum to TBEV can neutralize the infecting virus if applied in the early acute phase of the disease [101,334,335,336]. Several antiviral agents were also tested in cell cultures or mice infected with TBEV [337,338,339,340,341,342,343,344,345,346,347,348], and some of them seem promising (e.g., arbidol).

### 2.2. West Nile Virus (WNV; Genus: Flavivirus; Japanese Encephalitis Group)

Two strains of this mosquito-borne virus were isolated in Moravia from *Cx. pipiens* mosquitoes caught opposite to the Austrian village of Rabensburg in 1997 and 1999 [349,350]. The first isolate (97–103) was later sequenced and found to represent a new third genomic lineage of WNV [351]. Previously, only lineage WNV-1 was known in Europe before 1997. Another strain of WNV-3 was isolated later from *Ae. rossicus* in the same locality [352]. WNV-3 is less virulent than WNV-1, and it does not kill adult mice. The much more virulent lineage-2 WNV strains were recently recovered from *Cx. modestus* in south Moravia [353,354,355] and also in south Bohemia [356]. WNV could possibly persist in overwintering mosquitoes [357].

The vertebrate hosts are largely wild birds, and migratory birds play a role in the widespread geographic distribution of WNV [358]. VN antibodies to WNV were detected in the sera of 4 (out of 10 examined) wild geese (*A. anser*) in south Moravia [359,360]. Juřicová et al. [98,361] found HI antibodies to WNV in 9.7% of 295 passeriform birds caught in south Moravia. They also found WNV antibodies in 4 out of 82 passerine birds (14 spp.) in the Krkonoše mountains during the autumn migration [96] and in 5.5% of 273 house sparrows (*Passer domesticus*) caught in northern Moravia [97]. In south Moravia, 4.3% of the mostly wetland birds had antibodies to WNV [362]. This survey provided evidence of the local circulation of WNV in the littoral of the Nesyt fishpond near Mikulov, where many young wetland birds caught in July 1985 (hatched in that year locally) were seropositive without cross-reacting with TBEV. The possible local vector mosquito species were *Cx. modestus* or *Cx. pipiens*, which occur abundantly in the reed beds. The local WNV circulation was documented later when 11.3% of 574 domestic ducks living on the fishponds were seropositive for WNV in south Moravia [363]. Out of 31 cormorants (*Phalacrocorax carbo*) in south Moravia, 9.7% had antibodies to WNV [364]. In southern Moravia, 5.9% of 391 wild birds representing 28 species had PRN antibodies against WNV [365], including *Fulica atra*, *Alcedo atthis*, *Acrocephalus scirpaceus*, *A. schoenobaenus*, *A. palustris*, *L. luscinioides*, *Emberiza schoeniclus*, *Sylvia atricapilla*, *Remiz pendulinus*, *Parus caeruleus* and *Sturnus vulgaris*. The common coot (*F. atra*) has been suggested as a useful serological sentinel species for indicating the presence of WNV in the Czech Republic instead of chickens [366].

Many cases of free-living raptors and those in falconries (mainly goshawks) with lethal neuroinfections caused by the WNV-2 lineage were detected in Czech lands recently [367,368].

Of 93 wild boars in south Moravia, 6.5% were positive for WNV (PRN) antibodies [369]. The PRNT serosurvey was repeated in southern Moravia about 10 years later, with additional wild artiodactyl species being examined (105 *Capreolus capreolus*, 148 *Cervus elaphus*, 287 *Dama dama*, 71 *Ovis musimon* and 412 *Sus scrofa*). The overall seropositivity was 5.9%, with a range in individual species from 4.1% to 9.9% [370].

A serological survey (PRNT) for WNV in 163 horses in Czech lands was negative, but in neighboring Slovakia, 8.3% of 229 nonvaccinated horses were seropositive [371]. Later (2011–2013), a large serosurvey of 2349 horses found a specific WNV seropositivity of 0.7% among nonvaccinated equids in the Czech lands [372].

A serosurvey of the human population (*n* = 316) in three southern Moravían districts for WNV revealed only 0.6% positivity, but the sera were sampled in 1988–1999, which was prior the introduction of WNV in Moravia [373]. Sporadic human cases of WN disease have been diagnosed in south Moravia, with the first cases documented after the flood in 1997, which was followed by an enormous mosquito population [374,375]. WNV cases are still being reported in the Czech Republic [376,377].

Several contributions from Czech virologists to WNV diagnostics have been presented. CV-1 monkey cells and low-viscosity methylcellulose as an overlay were described for the plaquing and PRNT of WNV on plastic panels [285,286,287]. The preparation of hyperimmune mouse ascitic fluids and indirect IFA were described as useful tools for the identification and serodiagnostics of WNV [299,300].

Certain antiviral agents were tested on cell lines or mice infected with WNV [341,342,343,344].

### 2.3. Usutu Virus (USUV; Genus: Flavivirus; Japanese Encephalitis Group)

The vector of this African virus is mosquitoes (*Culex* spp., *Coquillettidia aurites*, *Mansonia africana*). In Czech lands, USUV was first isolated from birds [378] and then from *Cx. pipiens* and *Cx. modestus* mosquitoes [354,379].

The vertebrate hosts are birds, and the common coot (*Fulica atra*) has been suggested as a useful serological sentinel species for the indication of the presence of USUV in the area [370].

USUV is very pathogenic for certain passeriform birds (especially for the blackbird *Turdus* *merula* in Europe) and raptors [378,379].

Little is known about the medical importance of USUV. Rare human cases (usually in immunocompromised persons) have been reported in Europe, including in the Czech Republic [377].

## 3. Family: *TOGAVIRIDAE*

### Sindbis Virus (SINV; Genus: Alphavirus)

In the Czech Republic, antibodies to SINV have been found infrequently in south Moravia and south Bohemia [12,380,381,382,383,384,385].

The vectors of SINV are ornithophilic mosquitoes. The natural foci of SINV infections occur mainly in wetland ecosystems of diverse biomes (principally in an avian–mosquito cycle).

Vertebrate hosts are largely wild passeriform birds and occasionally rodents and amphibians.

Migratory birds play an important role in the wide geographic distribution of the virus. In south Moravia, 14.2% of 106 wild geese (*Anser anser*) were positive for SINV antibodies [360]. Juřicová et al. [361] found that 6.4% of 295 passeriform birds caught in south Moravia had SINV antibodies. Later, in the same area, only 0.7% of 178 passerine birds were seropositive for SINV [386]. SINV antibodies were found in 2.9% of birds in the family *Hirundinidae* (*n* = 183) [387]. Out of 31 cormorants (*Phalacrocorax carbo*) in south Moravia, 9.7% revealed antibodies to SINV [364]. Juřicová [96] examined 82 passerine birds of 14 spp. in the Krkonoše mountains during autumn migration and found antibodies to SINV in one robin (*Erithacus rubecula*). She also tested 273 house sparrows (*Passer domesticus*) caught in northern Moravia. SINV antibodies were present in 2.2% of them [97].

SINV antibodies have only been detected once in wild boars in south Moravia [369]. Kolman [380] found no seropositivity to SINV in domestic mammals including horses, cows and pigs living in south Moravia (district: Břeclav). However, the SINV antibodies were present in 1.0% of 104 hens and 8.5% of 47 ducks. Of 574 domestic ducks kept on 5 fishponds in south Moravia, only 0.3% were seropositive for SINV [363].

Recently, RNA of the Kyzylagach variant of SINV was detected by PCR in 4 of 221 pools prepared from 10,784 female *Culex modestus* mosquitoes collected at a fishpond in south Moravia [388].

Sluka [389] mentioned a febrile patient in Valtice who seroconverted against the Semliki Forest virus antigen (HI titer rose from negativity to 1:80). It is possible that the etiologic agent in this case was SINV due to the cross-HI reaction of SINV with the Semliki Forest virus.

CV-1 monkey cells and low-viscosity methylcellulose as an overlay were described for the plaquing and PRNT of SINV on plastic panels [285,286,287]. The preparation of hyperimmune mouse ascitic fluids and the immunofluorescence assay were described as useful for the serodiagnostics of SINV [299,300].

## 4. Family: *BUNYAVIRIDAE*

### 4.1. Ťahyňa Virus (TAHV; Genus: Orthobunyavirus; California Group)

TAHV was originally isolated (prototype strain Ť-92) from *Aedes vexans* and *Ochlerotatus caspius* mosquitoes in the village of Ťahyňa (eastern Slovakia) in 1958 [390]. This is the very first mosquito-borne virus that was isolated in Europe. In the Czech Republic, TAHV was first recovered from *Ae. vexans* in southern Moravia during 1962 [391]. A cross-neutralization study among the main viruses of the California group was carried out, showing a close relationship between TAHV and three North American agents: California encephalitis virus, LaCrosse virus and Snowshoe Hare virus [392].

The arthropod vectors of TAHV are mosquitoes. In the Czech lands (predominantly in southern Moravia), the virus was isolated from *Ae. vexans*, *Ae. cantans*, *Ae. caspius*, *Ae. sticticus*, *Ae. cinereus*, *Ae. dorsalis*, *Culex modestus*, *Culiseta annulata* and *Anopheles hyrcanus* [392,393,394,395,396,397,398,399,400,401,402,403,404,405,406,407,408,409,410,411,412,413,414]. These species of mosquitoes can be competent vectors, possibly also *Ae. excrucians*, *Ae. flavescens* and *Ae. communis* [398,411]. Rare TAHV isolations from mosquitoes are from northern Bohemia [414]. The dissemination of TAHV to various organs and body parts (legs) of *Ae. vexans* was followed experimentally [396,397,415]. Transovarial transmission of TAHV was documented in *Ae. vexans* [416] and *Cs. annulata* [417,418] as well as its overwintering in female *Cs. annulata* [419,420,421]. The seasonal occurrence of TAHV infections thus depends upon the seasonal activity of competent mosquito vectors [400,407]. One exception was the isolation of two strains of TAHV from ceratopogonid flies (*Culicoides*) in Moravia [422].

TAHV multiplies in mosquito cell lines (e.g., from *Aedes albopictus*) but without producing CPE [411,423,424]. Danielová [425] tested TAHV replication in the *Ae. albopictus* cell line at different temperatures. The virus grew in the range of 6–28 °C, more slowly at the lower temperatures, and it survived at 10 °C and 15 °C for more than 300 days.

The vertebrate hosts of TAHV in the Czech lands are mainly lagomorphs, hedgehogs and rodents. Antibodies or (experimental) viremia were demonstrated in rabbits and hares (*Lepus europaeus* and *Oryctolagus cuniculus*), hedgehog *Erinaceus concolor*, rodents (*Citellus citellus*, *Glis glis* and *Sciurus vulgaris*) and other mammals, including *Sus scrofa*, *Capreolus capreolus*, *Cervus elaphus*, *Martes foina*, *Putorius eversmanni*, *Vulpes vulpes* and *Myotis myotis* [411,426,427,428,429,430,431,432,433,434,435,436]. Hedgehogs inoculated with TAHV in winter (January) during the hibernation period showed viremia, and the virus could be isolated from organs up to 14 DPI [431]. For wild boars in south Moravia, 19.4% were positive for TAHV antibodies [369]. Serosurveys of game animals in south Moravia revealed seropositivity in a number of wild mammals [90,437].

Antibodies to TAHV were detected in 73.3% of domestic pigs in south Moravia in the Břeclav district [429]. Kolman [380] found high seropositivity to TAHV in domestic mammals living in that district, including 34.4% of 61 horses, 55.0% of 109 pigs and 5.6% of 305 cattle (but 49% of 346 local cattle were seropositive when tested in 1974 [411]). Antibodies for TAHV in domestic fowl were negligible at 1 of 104 hens and 0 of 47 ducks. Wild pheasants (*Phasianus colchicus*, 58 individuals) were seronegative [429], and when the pheasants were inoculated with TAHV, they did not reveal signs, viremia or antibodies [438]. In south Moravia, 106 wild geese (*Anser anser*) were examined for VN antibodies to TAHV, and 5.7% were positive [360]. However, no HI antibodies to TAHV were detected via the HIT in 280 wild birds of the 29 spp. living in the area [384]. Of 574 domestic ducks kept on 5 fishponds in south Moravia, 8.0% were seropositive in the HIT [365]. Juřicová et al. [361] tested via the HIT 295 passeriform birds caught in south Moravia, and TAHV antibodies were present in 16.3% of them. In the same area, 178 passerines were tested later, and 14.0% were seropositive [386]. Juřicová [96] examined 82 wild passerine birds (14 spp.) in the Krkonoše mountains during the autumn migration and found antibodies to TAHV in 9.8% of the birds. Out of 31 cormorants (*Phalacrocorax carbo*) from south Moravia, 22.6% revealed HI antibodies to TAHV [364]. Juřicová et al. [97] later tested 273 house sparrows (*Passer domesticus*) caught in northern Moravia, and TAHV antibodies were present in 14.7% of them. Additionally, in 4.4% of the birds of the family *Hirundinidae*, antibodies to TAHV were detected [387].

The formation of VN antibodies in mice infected with TAHV was influenced by the sex and inoculum dose [439]. The open-field activity of mice inoculated with TAHV changed, although obvious clinical signs were not observed [440]. Host immunosuppression with cyclophosphamide reverted the asymptomatic TAHV infection of mice into a lethal disease [441].

The effects of serial passaging of TAHV in Syrian hamsters and suckling mice and the cell cultures on the properties of the virus were studied [442]. Researchers found the appearance of small-plaque variants on GMK cells and virulence modifications (index of invasivity and average survival time in suckling mice). A similar study with 10 TAHV strains showed that after three passages in suckling mice, the virus titer increased, and the plaque size formation on GMK cells was reduced [443]. Málková [444,445,446,447] studied TAHV thermosensitivity and the effect of a higher temperature (39.2 °C) on TAHV in chick embryo cells. CPE started later and was less intense than that at 36.5 °C. CV-1 monkey cells and low-viscosity methylcellulose as an overlay were described for the TAHV plaquing and PRNT on plastic panels [285,286,287]. Málková [448] observed that carboxymethycellulose in the overlay increased the TAHV plaque number in GMK cells. Málková and Marhoul [449] tested the plaquing of several TAHV strains in GMK cells and found two TAHV variants with different plaque size virulence and thermostability values [450]. This was confirmed later when it was shown that specific strains of TAHV can differ in plaque size on vertebrate cell cultures and their antigenic properties, and their virulence can be affected by the number of passages in vitro [451]. The nucleotide variability of different strains of TAHV was documented, and the S segment was highly conservative in all analyzed TAHV strains. Within the M segment, the highest variability was observed in the G(C) gene encoding viral envelope protein and, to a lesser extent, in the NS gene as well [452,453].

The preparation of hyperimmune mouse ascitic fluids and indirect IFA were described as useful tools for the identification and serodiagnostics of TAHV [299,300]. For the HIT, stabilization of goose erythrocytes by formaldehyde and preparation of a high-titer hemagglutinin were suggested [454,455,456]. A modified plaque assay of TAHV in the GMK cells was reported [457]. A useful VNT on micropanels for the detection of TAHV antibodies was also described [458]. A nonspecific inhibitor of hemagglutination of TAHV in acetone-extracted mouse sera was detected, and its removal by acetone and heat was performed [459]. When the sensitivity of the PS cells, XTC-2 cells and suckling mice for the detection and isolation of California group bunyaviruses was compared, the mice were found to be the most sensitive [460].

The natural foci of TAHV disease occur in water-inundated lowland habitats (floodplain forest ecosystem) of the Czech lands, mainly situated in south Moravia [407,408,411,429,461,462,463]. High frequencies (up to 60–80%) of TAHV seroprevalence rates have been reported regularly in adult human populations in endemic south Moravian foci [251,375,429,464,465,466,467,468,469]. In the other parts of the Czech Republic, the TAHV seroprevalence rates in humans have been much lower, usually being below 10% [203,251,381,382,383,385,461,465,470,471].

Human disease caused by TAHV, called “Valtice fever” starting in 1960, is an influenza-like febrile illness occurring in the summer and early autumn mainly in children, with a sudden onset of fever (lasting 3–5 days), headache, dizziness, malaise, conjunctivitis, pharyngitis, myalgia, nausea, gastrointestinal disorders, loss of appetite, anorexia, occasional arthralgia, stiff neck or other signs of the CNS involvement (meningitis) and sometimes bronchopneumonia [8,457,472,473,474,475,476,477,478,479]. No lethal cases have been described for TAHV, contrary to the closely related North American La Crosse virus. At least 50 documented cases of Valtice fever in Moravia have been published since 1963 [461,466,472,473,474,475,476,477,478,479,480,481,482,483], but many cases have remained undiagnosed or unreported, since this is not a notifiable infectious disease in Europe. TAHV has been isolated from the blood of patients with Valtice fever several times [475,477,480,481].

Experimental infections of primates with TAHV yielded interesting results. Eighteen rhesus monkeys (*Macaca mulatta*) exposed to infectious aerosols did not develop a fever, other clinical signs (except for one animal), viremia or VN antibodies 28 DPI. When five chimpanzees (*Pan troglodytes*) were inoculated with TAHV s.c., they developed a fever for 3–4 days and an enhanced erythrocyte sedimentation rate and decreased mobility. Viremia lasted 3–8 DPI, and antibodies (HI, CF and VN) were formed 14 DPI [484,485,486]. Five chimpanzees were exposed to TAHV-infected *Cs. annulata* mosquitoes [487]. Fever and other symptoms were similar to those in the previous experiment.

### 4.2. Batai Virus (BATV; Genus: Orthobunyavirus; Bunyamwera Group; Synonym Čalovo Virus)

BATV was originally isolated from *Culex gelidus* collected in Kuala Lumpur (Malaysia) in 1955 [13]. The antigenically identical “Čalovo” virus (strain 184) was isolated from *Anopheles maculipennis* s.l. mosquitoes collected in a stable at Čalovo (village of Trstená in south Slovakia) in 1960 [488]. BATV was then also isolated from anopheline mosquitoes in the district of Břeclav in southern Moravia [406,489]. The genome of BATV strains was analyzed lately and revealed that Eurasian and African strains of BATV are phylogenetically different and form two distinct lineages [490].

The principal vectors of BATV in Europe are zoophilic mosquitoes. In south Moravia, BATV was isolated only from *An. maculipennis* s.l. collected in stables, and two strains from 10,901 anophelids were recovered [405,491].

The vertebrate hosts are horses, cattle and domestic pigs [8,380]. Antibodies to BATV were detected repeatedly in domestic animals in south Moravia. A high seropositivity to BATV was found in domestic mammals living in the district of Břeclav (27.9% of 61 horses, 25.2% of 305 cows (maintained in stables) and 17.4% of 109 pigs, but only 1.0% of 104 hens and 0.0% of 47 ducks [380]. Out of 574 domestic ducks kept on 5 fishponds in south Moravia, only 2.1% were seropositive for BATV [363]. Additionally, 106 wild geese (*Anser anser*) in south Moravia were examined, and 4.7% were seropositive [360]. Juřicová et al. [97] found BATV antibodies in 2.2% of 273 house sparrows (*Passer domesticus*) caught in northern Moravia. In south Moravia, 12.1% of 295 passeriform birds had BATV antibodies [95]. In the same area at a later date, 6.8% of 178 passerines were seropositive [286]. Juřicová [96] also examined 82 passerine birds of 14 spp. caught in the Krkonoše mountains during autumn migration and found antibodies to BATV in 3 birds.

The natural foci of BATV infection occur in agroecosystems (i.e., in and around villages and farms), and principally, virus circulation is based on the “domestic animal–zoophilic mosquito” cycle.

BATV reproduces (with the formation of CPE) in CEC, BHK-21 and PS cells [423]. It also multiplies (without CPE) in *Anopheles stephensi* and *Aedes albopictus* mosquito cells, but it does not multiply in cell line Mos 55 from *An. gambiae* [424,490].

The virus is pathogenic for suckling and juvenile mice (after i.c. and s.c. inoculation) and chick embryos but not for adult rats, guinea pigs and chickens [8,12].

BATV could be pathogenic for other mammals. In humans, seroconversion data have indicated an association of BATV with influenza-like illness accompanied by malaise, myalgia and anorexia in south Moravia [389,474,492,493]. Further research on the pathogenic role of BATV in humans is necessary.

### 4.3. Lednice Virus (LEDV; Orthobunyavirus; Turlock Group)

The prototype strain 6118 of LEDV was isolated from *Cx. modestus* mosquitoes collected in the reedbeds of fishponds at Lednice in south Moravia (Czech Republic) in 1963 [405] and identified later [494]. Six additional isolations were made at the same locality in 1972 [409]. The virus was originally classified as the Yaba-1 (=M’Poko) virus of the Turlock antigenic group [495], which is known to occur in Africa. The LEDV genome was later sequenced, and it was found that the virus is related to but different from the Umbre, Turlock and Kedah viruses of the Turlock group [496].

Some physical and chemical properties of the virus (e.g., the effect of pH, temperature, HA formation and morphology) were described [497,498], as well as biological properties such as the pathogenicity (mice, rats, golden hamsters, guinea pigs and chickens are all refractory) [499] in LEDV replicates in chick embryo [500] and chick embryo cells [501].

The only known competent vector is *Cx. modestus* [12,502]. The virus does not replicate in cell line Mos-55 from *An. gambiae* [424].

The vertebrate hosts are wetland birds, largely of the order *Anseriformes*. In 1975–1977, antibodies were detected in south Moravian fishponds [12,384,503] in *Cygnus olor* (2/4 seropositive), *Anser anser* (14/95 seropositive) and *Anas platyrhynchos* (22/69 seropositive). LEDV antibodies were also detected in domestic fowl bred at fishponds in the endemic area in 1972 [381] and in 1975–1977 [384] in geese (7/185 and 10/436) and ducks (25/141 and 21/295), but in hens the rates were 0/100 and 1/208, respectively. Kolman et al. [384] examined 280 wild birds of 29 spp. living in the endemic area and detected antibodies to LEDV in anseriform birds living on the fishponds with reed beds (i.e., the habitat of vector *Cx. modestus*), namely *A. platyrhynchos* (22/69), *A. anser* (11/64) and *C. olor* (1/3), while no specimens were seropositive of the 65 passeriform birds caught in the same ecosystem (*Acrocephalus* and *Locustella* spp. and *Emberiza schoeniclus*). The experimental viremia in several local wild bird species, including *Phasianus colchicus*, *Larus ridibundus* and *Fulica atra*, was generally low and of a short duration [12,504,505]. Domestic chickens, ducklings and goslings inoculated s.c. with LEDV did not develop signs of illness, and the viremia was short and of a low level. VN antibodies appeared after 2–3 weeks post-inoculation [506,507]. LEDV multiplies in chick embryos and causes histological lesions in the brain, striated muscles and heart and vascular endothelium [12,499,500,508].

No disease signs, viremia or antibodies have been detected in local mammals (e.g., rodents, insectivores or deer) or in s.c. inoculated *Macaca mulatta* monkeys [509]. LEDV is fatal to suckling mice (inoculated i.c. and i.n. but not i.p. and s.c.) and partially pathogenic to adult mice (i.c. and i.n.) and nonpathogenic to suckling and adult rats, guinea pigs, golden hamsters, rabbits, ferrets and chickens [12,510]. A serosurvey of local domestic mammals (461 cows, 181 pigs, 91 horses, 6 goats and 50 sheep) resulted in a single positive cattle, but the HI titer was low (1:20) [511]. Forty-four local wild rodents (*Micromys minutus*, *Apodemus sylvaticus*, *Myodes glareolus* and *Microtus arvalis*) were all seronegative [12,510].

LEDV produces CPE in chick (duck and goose) embryo cells but not in mammalian cell lines (e.g., PS, Vero or CV-1) [12,499,501] or in mosquito cell lines from *Aedes aegypti*, *Ae. albopictus*, *Anopheles stephensi* or *An. gambiae* [423,424,490].

A good HA antigen of LEDV was prepared using ultrasonic waves [512]. Málková et al. [513] proposed radial hemolysis in gel as a useful serological test for LEDV.

Disease in vertebrates is unknown but quite improbable in mammals including humans, as no antibodies were detected in 581 inhabitants of the endemic area [464,511].

### 4.4. Sedlec Virus (SEDV; Genus: Orthobunyavirus; Simbu Group)

The prototype strain AV172 of the virus was isolated from the blood of a reed warbler (*Acrocephalus scirpaceus*) caught on the Nesyt fishpond near the village of Sedlec (close to Mikulov) in south Moravia on 30 July 1984 [362,514]. SEDV was identified as a member of the Simbu group of bunyaviruses [515,516].

The virus causes no symptoms in suckling or adult mice when given s.c., but for i.c. inoculation the virus kills them. Adult rabbits are not susceptible to SEDV and do not produce VN antibodies after i.v. inoculation. CPE is formed in CV-1 monkey cells (but not in Vero cells) and in SPEV pig cells, but only at an elevated temperature of 40–41 °C (i.e., at the avian body temperature).

Among 109 birds of 6 species caught on the Nesyt fishpond wetland in 1988, 25 (i.e., 22.9%) had VN antibodies against SEDV (titer of 1:10 or higher) when tested on CV-1 cells, namely the reed warbler *A. scirpaceus* (19/89), sedge warbler *A. schoenobaenus* (3/12), Savi’s warbler *Locustella luscinioides* (2/2), and reed bunting *Emberiza schoeniclus* (1/3) [514]. All these species live in reed beds of fishponds.

Arthropod vectors are still unknown, but possible vectors can be ornithophilic ceratopogonids.

Disease in humans or animals has not been described; serosurveys of humans and other mammals have not been carried out.

### 4.5. Uukuniemi Virus (UUKV; Genus: Phlebovirus)

UUKV was originally isolated from *I*. *ricinus* ticks collected from cattle near the village of Uukuniemi in southeast Finland in 1959. In 1963, an agent named the “Poteplí” virus was isolated from *I*. *ricinus* in Central Bohemia [517] and at an MIR of 3.95, while the MIR of TBEV in the *I. ricinus* population was only 1.91 in this locality [233]. This virus was later found to be antigenically identical with the Finnish UUKV. The biological and physico-chemical properties of the virus were studied [518,519]. UUKV is not pathogenic for adult mice when given s.c. or i.p.

In addition to the Poteplí locality, UUKV was isolated from *I*. *ricinus* ticks in northern Moravia [520,521], urban parks in Prague [217,219,233] and southern Moravia [248].

The arthropod vectors are ixodid ticks, and TST of UUKV occurs in *I. ricinus* [522].

The vertebrate hosts are forest rodents (*Myodes glareolus* and *Apodemus flavicollis*) and birds, largely ground-feeding passerines. Kolman and Husová [88] examined via CFT small mammals (288 *A. flavicollis*, 161 *M. glareolus* and 101 *Microtus arvalis*) in a natural focus of TBE at Unhošť in central Bohemia, and antibodies to UUKV were present in the three species (3.7%, 1.2% and 0.0%, respectively).

Fatal meningoencephalitis with myositis occurs in suckling mice, but no symptoms have been observed in adult mice via any route of inoculation or in adult rats (i.c.). UUKV is also pathogenic to suckling but not adult *M. arvalis*, *A. flavicollis* or *M. glareolus* (i.c., but usually not i.p.) or suckling rats (i.c., but not i.p.). UUKV is non-pathogenic for rhesus monkeys (i.p.).

Only partial CPE (or no CPE) is formed by UUKV in several primary mammalian cells (e.g., kidney cells from susliks, dormice and martens) [523], but CPE is produced in BSC-1, BHK-21 and PS cells. UUKV replication does not occur in *Aedes egypti*, *Ae. albopictus* or *Ae. stephensi* mosquito cells [423,424,490].

Animal and human disease caused by UUKV has never been reported. Antibodies were detected in less than 5% of the persons examined in a few areas [524,525]. For serological diagnosis, CFT, HIT, IFA or VNT can be used [12].

## 5. Family: *REOVIRIDAE*

### Tribeč Virus (TRBV; Genus: Orbivirus; Kemerovo Group)

The prototype strain is Tribeč (*Ixodes ricinus*, west Slovakia, 1963). Its synonymous strains are Lipovník (*I. ricinus*, east Slovakia, 1963) and Cvilín (*I. ricinus*, north Moravia).

TRBV is closely related to the Siberian Kemerovo virus by CFT but is distinguishable by VNT. The gene pools of the Kemerovo group and other orbiviruses have great reassortment potential (because of the segmented dsRNA genome) and resulting biological variability.

The principal vector for TRBV is the *I. ricinus* tick. In the Czech lands, TRBV or its variants were isolated from *I. ricinus* in Cvilín (district of Bruntál) in 1975 [526], Tetčiněves (district of Litoměřice) in 1976, Sychrov (district of Liberec) in 1976, Chuchelná (district of Opava) in 1978 and Černá Voda (district of Šumperk) in 1978 [527].

The vertebrate hosts of TRBV are rodents, hares and goats. Antibodies are often present in the grazed ruminants in endemic areas. Animal disease is unknown (inapparent infections); however, TRBV is fatal to suckling mice (also when inoculated s.c., showing meningoencephalitis with perivascular infiltration, progressive neuronal and glial damage). TRBV is fatal in suckling rats and suckling Syrian hamsters (i.c., but not s.c.). Meningitis with survival or no symptoms at all occur in adult mice under i.c. inoculation (but with local necrotizing encephalitis demonstrated histologically), while no symptoms are present in adult mice given s.c., i.n. or p.o. inoculation. Adult rats (i.c.), rabbits (i.c.) and peripherally inoculated calves or foals are also asymptomatic. Fever and meningitis are present in rhesus monkeys under i.c. inoculation. The CPE or plaques are produced in Hep-2, L (mouse) and RU-1 cells.

The virus occasionally causes febrile illness or aseptic meningitis in humans. A study reported at least 15 patients with the CNS infection (meningitis) and seroconversion against TRBV in the Czech lands. Seven of 154 patients hospitalized with meningoencephalitis (all negative for TBE) in central Bohemia presented IF antibodies to TRBV. Five of these sera were examined in PRNT, and although the titers were low, one serum reacted at titers of 1:16–1:32 against TRBV, suggesting seroconversion [525,528,529]. In northern Moravia, out of 49 patients with TBE-like symptoms, 16% revealed seroconversion or a significant increase in the PRN antibody titer to TRBV, and 7–8% of the healthy forest workers had antibodies to TRBV [527], while 9% of the 103 healthy adult residents in that region had TRBV antibodies as detected by PRNT [530]. A total of 4.9% of 408 persons with neuropathies in Central Bohemia had IF antibodies to TRBV, and 6 patients with meningoencephalitis seroconverted against TRBV [12]. In addition, Fraňková et al. [528] observed a meningoencephalitis case in central Bohemia caused by an orbivirus that was likely TRBV. In the Znojmo district hospital (southern Moravia), 19% of 75 febrile patients were seropositive to TRBV by PRNT. One patient out of 6 with meningoencephalitis and 10 out of 32 patients with meningitis had TRBV infections. In the same study of 28 forest workers in the same district, 22 had antibodies to TRBV by PRNT [531]. Additionally, in West Bohemia, antibodies to TRBV were detected in humans [203].

Interestingly, antibodies to TRBV were detected at a higher frequency among patients with multiple sclerosis [530]. However, additional studies are necessary to assess the public health importance of TRBV.

The HIT cannot be used in diagnostic serology, as TRBV does not produce hemagglutinin [12,528].

## 6. Conclusions

Czech researchers and medical doctors (clinicians and epidemiologists) have contributed significantly to the study of these arboviruses and the diseases they cause since the year 1948.

Three tick-borne viruses (“tiboviruses”) have occurred in the Czech lands since the 20th century: TBEV, UUKV and TRBV. Their recognized vertebrate hosts are rodents, insectivores and certain other mammals. The most important tibovirus, TBEV, causes serious human disease, usually biphasic fever with a rapid onset, sweating, headache, nausea, weakness, myalgia and arthralgia. Frequently, there are CNS affections, including meningitis, meningoencephalitis or encephalomyelitis with paresis, paralysis and other neurological sequelae. TRBV might play a role in human or animal pathology, though the disease is usually either less serious or infrequently reported. UUKV is an “orphan” virus without proven medical or veterinary significance. However, certain arboviral diseases of vertebrates may often pass unnoticed or be misdiagnosed, and they might potentially appear to be emerging diseases.

Seven mosquito-borne viruses (“moboviruses”) have occurred in the Czech Republic since the 20th century: WNV, USUV, SINV, TAHV, BATV, LEDV and SEDV (however, the last virus is probably transmitted by biting midges). The recognized or potential vertebrate hosts of these moboviruses are insectivores (TAHV), bats (SINV, WNV and TAHV), rodents (SINV, WNV, BATV and TAHV), lagomorphs (TAHV), carnivores (BATV and TAHV), equids (WNV (BATV)), artiodactyls (WNV and BATV (TAHV)), primates (SINV, WNV, BATV and TAHV), birds (SINV, WNV, USUV, BATV, LEDV and SEDV) and amphibians (SINV and WNV). Out of these moboviruses reported in the Czech lands to date, five can cause human disease (WNV, USUV, SINV, TAHV and BATV), and two are associated with birds and are probably not pathogenic to humans (LEDV and SEDV). Two groups of mobovirus diseases can be recognized according to the main clinical symptoms produced: (1) febrile illness with sweating, headache, nausea, weakness, myalgia, arthralgia and rash and (2) CNS affection with meningitis, meningoencephalitis or encephalomyelitis with paresis, paralysis and other neurological sequelae. Mobovirus outbreaks are strictly determined by the presence or import of particular competent vectors of the disease. Ecological variables (including weather and climate conditions) affect moboviruses considerably. The main factors are an abundance of mosquito vectors and their vertebrate hosts, intense summer precipitations, floods, higher summer temperatures and drought and the presence of appropriate habitats.

## Abbreviations

CF(T): complement fixation (test); CPE: cytopathic effect; CSF: cerebrospinal fluid; DPI: days post inoculation; HA: hemagglutinin; HI(T): hemagglutination inhibition (test); i.c.: intracerebral; IF(A): immunofluorescence (assay); i.m.: intramuscular; i.n.: intranasal; i.p.: intraperitoneal; i.v.: intravenous; MIR: minimum infection rate (per 1000 examined individual mosquitoes or ticks); p.o.: peroral; PRNT: plaque reduction neutralization test; s.c.: subcutaneous; TBE: tick-borne encephalitis; TOT: transovarial transmission (in arthropods); TST: transstadial transmission (in arthropods); VN(T): virus neutralization (test).
